# The Importance of Endoscopy with Biopsy: Real-World Evidence of Gastrointestinal Involvement in Primary Immunodeficiency in Two Main Northern Italian Centres

**DOI:** 10.3390/biomedicines11010170

**Published:** 2023-01-10

**Authors:** Stefania Nicola, Francesco Cinetto, Stefano Della Mura, Luca Lo Sardo, Elena Saracco, Ilaria Vitali, Riccardo Scarpa, Helena Buso, Vera Bonato, Claudia Discardi, Giovanni Rolla, Carla Felice, Marcello Rattazzi, Luisa Brussino

**Affiliations:** 1 Allergy and Clinical Immunology Unit, Department of Medical Sciences, University of Torino & Mauriziano Hospital, 10128 Torino, Italy; 2 Department of Medicine-DIMED, University of Padova, 35122 Padua, Italy; 3 Rare Diseases Referral Center, Internal Medicine I, Ca’ Foncello Hospital, AULSS2 Marca Trevigiana, 31100 Treviso, Italy

**Keywords:** inborn errors of immunity, IEI, common variable immunodeficiency CVID, chronic enteropathy, nodular lymphoid hyperplasia, NLH, colonoscopy, histology, gastrointestinal symptoms, intestinal biopsy, celiac-like disease

## Abstract

Introduction: Inborn errors of immunity (IEI) represent a heterogeneous group of diseases in which the true prevalence of GI involvement is not well-known. This study evaluates the prevalence of lower GI manifestations in patients with common variable immunodeficiency (CVID), analysing the histologic findings in colonic samples and assessing any correlations with biochemical abnormalities. Materials and Methods: A retrospective study was performed by collecting the data of IEI adult patients followed up at two main Northern Italian centres. Demographic and clinical data, and blood tests were collected. A colonoscopy with multiple biopsies in standard sites, in addition to a biopsy for any macroscopic lesion, was performed. The gastrointestinal Symptom Rating Scale for Irritable Bowel Syndrome (GSRS-IBS) and the short Inflammatory Bowel Disease Questionnaire (sIBDQ) were used to assess GI symptoms. Results: 141 patients were included: 121 (86.5%) with CVID, 17 (12.1%) with IgG subclass deficiency, and 2 (1.4%) with X-linked agammaglobulinemia. Of the patients, 72 (51%) complained of GI symptoms. No differences were seen between patients receiving or not IgRT. GI infections were found in 9 patients (6.4%). No significant correlations were found between gut infections and symptoms or leukocyte infiltrates. Colonoscopy alterations were present in 79 patients (56%), and the most common were colon polyps (42%). Microscopical abnormalities were seen in 60 histologic samples (42.5%) and the most frequent was nodular lymphoid hyperplasia (40%). A leukocyte infiltrate was present in 67 samples (47.5%), and the most common was a lymphocyte infiltrate (33%). No correlation was found between GI symptoms and macroscopic alterations, whereas a positive correlation between symptoms and microscopic alterations was detected. Conclusions: GI symptoms and microscopic alterations in colon samples are closely related; hence, it is important to carry out serial colonic biopsies in every CVID patient, even in the absence of macroscopic lesions.

## 1. Introduction

Inborn errors of immunity (IEI) represent a heterogeneous group of diseases characterised by defects in either cellular or humoral immunity [1]. Common variable immunodeficiency (CVID) is the most common symptomatic IEI among adults, and its prevalence is estimated to be between 1/125,000 and 1/50,000, with important geographic gaps [2,3]. In support of this, many authors suggested that economic and psychosocial burdens are related to geographical disparities. Several studies showed that the economic impact of the disease before diagnosis could be responsible for an underestimation of IEI prevalence [4,5]. Thus, the prevalence ranges from 1.3–5/100,000 in Europe [2], 0.25/100,000 in Japan [6], and 30.2/100,000 in the USA [7]. 

According to their clinical presentation, patients with CVID could be classified as belonging to one out of five clinical phenotypes [8], and despite most having increased infections [9,10], up to 60% of CVID patients show some sort of gastrointestinal (GI) manifestations, including bloating, abdominal pain, and diarrhoea [11].

Important causes of GI symptoms in patients with CVID, especially in those with low IgA levels, are represented by chronic infections [12], including *Giardia lamblia, Campylobacter jejuni*, and *Norovirus* [13]. Among noninfectious abnormalities [14], autoimmune enteropathy [13], including nodular lymphoid hyperplasia and lymphocytic colitis [15,16], and malignancies [17] are frequently observed.

However, the true prevalence of GI involvement in patients with CVID is not yet well-defined. Our study, therefore, aimed at evaluating the prevalence of lower GI manifestations in a cohort of adult patients with CVID, analysing the different types of inflammatory infiltrates coming from biopsies of the colon and assessing any correlations with biochemical abnormalities.

## 2. Materials and Methods

### 2.1. Study Design and Participants

The study was an Italian retrospective study of adult IEI patients followed up at the Allergy and Clinical Immunology University Clinic in Turin (A.O. Ordine Mauriziano di Torino) and at the Immunologic Rare Disease Centre of Treviso (Ospedale di Treviso, ULSS 2 Marca Trevigiana). 

All the patients with a CVID, XLA, or subclasses of IgG deficiency diagnosis according to the Criteria of the European Society for Immunodeficiency (ESID) [1] who had had a total colonoscopy with intestinal biopsy performed within the last 5 years were enrolled in the study. Data were collected at same time as that of the colonoscopy.

Patients with secondary immunodeficiency, patients treated with intestinal corticosteroids or immunosuppressive drugs, patients with a prior diagnosis of celiac-like disease, and patients not releasing their informed consent were excluded from the research. Inclusion and exclusion criteria are summarised in Table 1.

This study was approved by the local ethics committee (*Comitato Etico per la Sperimentazione Clinica delle Province di Treviso e Belluno*, study number 818/CE Marca) and conducted according to the Declaration of Helsinki. Data were collected between September 2018 and February 2022, and all the enrolled patients released their informed consent.

### 2.2. Data Collection

Data concerning age, sex, smoking habits, ongoing treatments, GI symptoms, cancer prevalence, death and comorbidities, systemic or organ-specific autoimmune involvement, the presence of splenomegaly, granulomatous and lymphocytic interstitial lung diseases (GLILD), and bronchiectasis are listed.

In addition, blood-test, colonoscopic, and histologic data were collected.

### 2.3. GI Symptoms Assessment

GI symptoms such as anorexia, nausea, vomiting, diarrhoea, abdominal pain, and discomfort were assessed using the Gastrointestinal Symptom Rating Scale for Irritable Bowel Syndrome (GSRS-IBS) and short Inflammatory Bowel Disease Questionnaire (sIBDQ) [18]. 

### 2.4. Blood and Lab Tests

All patients were taken for blood tests as a part of the routine follow-up. Complete blood count, IgG, IgA, IgM, and IgG subclass level at diagnosis, and faecal samples analysis for microbial detection (stool sample culture test, parasitological examination, and RT-PCR assay for *Giardia lamblia* and *Blastocystis hominis*) were collected from all participants [19,20]. T- and B-lymphocyte subsets according to the EUROclass trial [21,22], including IgD-IgM-CD27^+^ switched memory B cells (SM B cells), and CD21^low^CD38^low^ (activated B cells) were also recorded. In addition, for patients receiving immunoglobulin replacement therapy (IgRT), serum IgG at the trough level (IgG TL) was also stored.

Blood tests and faecal cultures had both been performed before the patients underwent the colonoscopy.

### 2.5. Endoscopic Examination and Histopathological Assessment

All patients underwent colonoscopy with multiple biopsies in standard sites (ascending, transverse, and descending colon), in addition to a biopsy for any macroscopic lesion. The assessed macroscopic features included haemorrhoids, polyps, diverticula, mucosal oedema, and erosions [6].

Intestinal biopsies were collected at the department of gastrointestinal endoscopy of S.C. Gastroenterologia A.O. Ordine Mauriziano di Torino and U.O.C. Gastroenterologia Ospedale di Treviso-ULSS 2 marca Trevigiana, and analysed at the division of Anatomic Pathology of S.C. Anatomia Patologica A.O. Ordine Mauriziano di Torino and U.O.C. Anatomia e Istologia Patologica Ospedale di Treviso ULSS 2 marca Trevigiana. 

Tissue samples were fixed in formaldehyde and then embedded in paraffin prior to staining with hematoxylin–eosin and Giemsa [23].

The assessed histology features were the presence of nodular lymphoid hyperplasia (NLH), cryptitis, cryptic abscesses, crypt distortion or increased inflammatory cells in the lamina propria, lymphocytic colitis, collagenous colitis, and acute colitis [13]. Pathologists also searched for inclusion bodies of human cytomegalovirus (CMV) in all samples.

Concerning cellular mucosal infiltrate, the cut-off level for increased intraepithelial lymphocytes in colonic biopsies was ≥20 lymphocytes per 100 surface epithelial cells [24], whereas the thresholds for neutrophils, eosinophils, and plasma cells [25] were 0 neutrophils/HPF, >100 eosinophils/HPF in the right colon, >84 eosinophils/HPF in the transverse and descending colon, >64 eosinophils/HPF in the rectosigmoid colon, respectively [26].

### 2.6. Statistical Analysis

Statistical analysis was performed using the IBM © SPSS Statistics for Windows package, version 26.0 (IBM Corp., Armonk, NY, USA). First, the normality distribution of data was tested using the Kolmogorov–Smirnov normality test, and a descriptive analysis of the variables was then performed. Baseline characteristics were evaluated in the whole cohort and, unless specified, expressed as mean (±SD) for continuous variables, and as absolute and relative frequencies for categorical variables. 

The analysis was performed using parametric (Student’s *T*-test) or nonparametric methods (Mann–Whitney’s U and Kruskal–Wallis tests) for continuous variables, and with χ2 tests and Fisher’s exact test for categorical variables. The *p*-values below 0.05 were considered to be statistically significant.

## 3. Results

A total of 141 patients (75 females, 53.5%), with a mean age of 54.14 years (range, 23–83) were included in the study. Of them, 121 (86.5%) patients had a diagnosis of CVID, 17 (12.1%) showed IgG subclass deficiency, and 2 (1.4%) were diagnosed with X-linked agammaglobulinemia (XLA). In addition, 101/141 (71.6%) patients received IgRT: among CVID patients, 86/121 (71%) were receiving IgRT, and 13/71 (76.5%) among IgG subclass deficiency and 2/2 (100%) among XLA.

Because of the small cohort numbers, and the severity of gastrointestinal manifestations and infections reported by the patients with a IgG subclass deficiency and XLA, we decided to merge them with CVID patients and to consider all IEI patients as a single group for statistical analysis.

Regarding the smoking habit, 94 patients were nonsmokers, 21 were active smokers, 23 were former smokers, and 3 did not state their smoking habits.

Baseline characteristics of the enrolled sample, including demographic data, comorbidities, complete blood count, lymphocyte subsets, and immunoglobulin levels at diagnosis and at the TL are summarised in Table 2 and Table 3.

### 3.1. Prevalence of Gastrointestinal Manifestations

Of the patients, 72 (51%) complained of some sort of gastrointestinal discomfort: 53 (74%) showed abdominal pain and diarrhoea with mean bowel movements of 4 ± 3/day, 2 (2.7%) patients had haematochezia, and 17 (23.6%) bloating and dyspepsia. In terms of GI symptoms, no differences were seen between patients receiving IgRT or not (data not shown).

### 3.2. Gastrointestinal Infections

Nine patients (6.4%) had GI infections: Giardia lamblia was found in 6 patients (66.6%), whereas Blastocystis hominis was detected in 3 of them (33.3%).

No significant correlations were found between gut infection and symptoms or leukocyte infiltrates (data not shown).

### 3.3. Endoscopic Findings

Of the patients, 79 (56%) showed at least an appreciable alteration to colonoscopy, with colon polyps being the most frequent (42%), followed by haemorrhoids (16.5%) and mucosal oedema (11.4%), as shown in Table 4.

In addition, microscopic abnormalities were seen in 60 histologic samples (42.5%) and NLH was the most common finding, as it was highlighted in 24 cases (40%) (Table 4). 

Furthermore, a microscopic alteration in colon histological specimens was observed in 20 patients despite the absence of macroscopic lesions.

A leukocyte infiltrate was present in 67 analysed samples (47.5%), and the most common was a lymphocyte infiltrate (33%), followed by plasma cells (22.4%), eosinophils (14.9%), and neutrophils (13.4%). 

In addition, no CMV inclusion bodies are reported in the histologic samples of our population.

### 3.4. Correlations between Symptoms and Abnormalities

No correlation was found between gastrointestinal symptoms and macroscopic alterations to colonoscopy (data not shown).

A positive correlation between symptoms and microscopic alterations in the histologic samples was instead detected in the histologic samples (χ^2^ = 11.67, *p* = 0.001).

Among patients without symptoms (*n* = 69; 49.6%) the most common findings were no alterations (50/69; 74.4%) followed by tubular adenoma (9/69; 13%) and NLH (8/69; 11.6%) whereas in patients complaining of abdominal pain (*n* = 51; 36.7%) the most frequently observed findings were no alterations (22/51; 43.13%) followed by NLH (13/51; 25.5%) and tubular adenoma (8/51; 15.7%).

The distribution of microscopic abnormalities in colon biopsies is reported in Table 5.

### 3.5. Comparison between Patients with and without NLH

Patients with NLH showed significantly lower levels of circulating WBC, total lymphocytes, and T cells CD3+, compared to patients without NLH (*p* < 0.05, see Table 6). NLH patients also had lower levels of T cells CD3+/CD4+, CD21^low^CD38^low^ B cells and higher levels of SM B Cells, although the differences were not statistically significant. No differences were found between the two groups in terms of CD19+ B cells and immunoglobulin levels at diagnosis or at trough level (Table 6).

## 4. Discussion

This is the first real-world Italian study assessing gastrointestinal involvement in a large cohort of adult primary immunodeficiency patients. Not only did this study evaluate the gastrointestinal symptoms, but it also correlated them with the endoscopic findings and blood tests. This provides more evidence about the need for lower endoscopy screening with serial colonic biopsies in all CVID patients. 

Many studies [17,18,21,23,24,25,26] recommend the importance of screening colonoscopies every 5 years in these patients, but in clinical practice, serial colon biopsies are not routinely performed.

The prevalence of gastrointestinal symptoms in our cohort was about 50%. In accordance with other research [11], and abdominal pain, diarrhoea, haematochezia, and dyspepsia were the most common. At the same time, also in the literature, the most frequently reported lower GI presentations in CVID patients included diarrhoea, malabsorption, inflammatory bowel disease, nodular lymphoid hyperplasia, and infections [27].

In terms of infections, a few more than 5% of our patients showed a sort of intestinal infection, mainly due to *Giardia lamblia* and *Blastocystis hominis*. As expected, Giardia lamblia was the most relevant intestinal pathogen in CVID despite the prevalence in our study being far lower than that in other studies [12]. In a European cohort of patients with CVID, the prevalence of gastrointestinal parasitic or bacterial infections was 26.5% [28], and in a large North American study, 24% of patients had GI infections, with the most commonly identified pathogen being, Giardia lamblia [29]. 

However, many studies did not distinguish between acute and chronic infections, and most analysed the whole gastrointestinal system infection pattern. This means that upper GI infections were also considered, including *Helicobacter pylori* colonisation, which is estimated to affect up to 20% of CVID patients [28,29].

Hence, the relatively low prevalence of GI infections in our population might be explained by the study design. Our study focused therefore on chronic rather than acute infections and aimed at evaluating only lower GI involvement.

Regarding endoscopic abnormalities, about one-third of our patients showed colon polyps, in line with other research [28,30], whereas mucosal erosions were found in less than 5%, a lower prevalence compared with other large cohort studies [28,31].

Concerning the histologic assessment, we found a lymphocyte infiltrate in a large percentage of patients, like in many other studies. On the other hand, the prevalence of neutrophil infiltrate was lower than that reported in the literature [15]. However, neutrophil infiltrates are related to the epithelial damage caused by acute infections, including CMV and cryptosporidium, or chronic inflammation, such as inflammatory bowel disease (IBD)-like diseases [13,15]. 

In our cohort, we found a low prevalence of mucosal erosions and GI infections, including the lack in CMV inclusion bodies. These findings may, therefore, support the limited neutrophil infiltrate in our patients.

Besides the mere analysis of symptoms prevalence, one of the strengths of our study was the assessment of the correlations between symptoms and endoscopic alterations.

The analysis of the lower GI histological alterations in relation to the patients’ symptoms allowed for us to deserve several significant clinical implications. 

Among our patients, no correlations were found between symptoms and macroscopic alterations. Despite this, microscopic alterations were found in a relevant percentage of patients, with tubular adenomas and NLH being the most common ones. Since adenomas are seldom at high risk of malignancy progression, and NLH was hypothesised to be a risk factor for intestinal lymphoma development [32], our findings underline the importance of also always performing colonic biopsies in patients with even only mild/moderate or without GI symptoms. Moreover, as most patients with a normal endoscopic assessment already had microscopic abnormalities, our study indicates the need for always performing multiple colonic histologic samples in all CVID patients, independent of the macroscopic aspect.

Lastly, CVID patients with NLH showed many haematological abnormalities compared to those without NLH. As described in the literature, NLH can be associated with low levels of CD19+ B cells in tissue [13] despite a high number of CD19+ circulating B cells [11]. This is in line with our findings: CVID patients with NLH in our cohort had higher levels of CD19+ circulating B cells compared to those of patients with no NLH, although there was no statistical difference, possibly due to the limited cohort sample. 

Moreover, the slightly lower levels of CD21^low^CD38^low^ B cells that we found in CVID patients with NLH compared to patients without NLH could be linked to the impairment of memory B cells. This might promote tissue lymphoid hyperplasia, but other studies are needed to investigate the underlying mechanism [33]. 

Of growing interest in this field is the relationship between the intestinal microbiome and IEI, as different clinical manifestations of these disorders could be related to a loss of microbiome diversity and dysbiosis [34]. Furthermore, the diet could play an important role in malignant progression, considering the ability of amino acids to modulate immune system regulation [35,36,37].

However, this study has some limitations to consider. First, the results are based on retrospective data gathered from clinical history and medical records. Moreover, a selection bias might also be present due to the enrolment of patients coming from only two main centres in Northern Italy.

## 5. Conclusions

In summary, GI involvement in CVID patients is very common and may not always be associated with the presence of GI symptoms. Our pilot study highlighted the lack of correlations between GI symptoms and the macroscopic alterations at colonoscopy, whereas GI symptoms and microscopic alterations in colon samples are closely related. Hence, the importance of carrying out serial colonic biopsies in every CVID patient becomes increasingly clear, even in the absence of macroscopic lesions, to early diagnose any malignant progression. Further studies are needed to evaluate the risk factors involved in the occurrence of malignancy in CVID patients. 

## Figures and Tables

**Table 1:** Inclusion and exclusion criteria.

Inclusion Criteria	Exclusion Criteria
CVID, XLA, or subclasses of IgG deficiency diagnosis according to the Criteria of the European Society for Immunodeficiency (ESID).	Secondary immunodeficiency.
Total colonoscopy with intestinal biopsy performed within the last 5 years.	Treatment with intestinal corticosteroids or immunosuppressive drugs.
	Prior diagnosis of celiac-like disease.
	Informed consent not granted.

**Table 2:** Demographic data and comorbidities at baseline.

Patients	*n* = 141 (%)
*Demographic data*	
Sex	
M	66 (46.5)
F	75 (53.5)
Age (years)	54.14 (range 23–83)
Smoking habits	
Nonsmokers	94 (66.6)
Active smokers	21 (14.8)
Former smokers	23 (16.3)
Not known	3 (2.1)
*Comorbidities*	
Bronchiectasis	54 (38.2)
Splenomegaly	35 (24.8)
Granulomatous lymphocytic interstitial lung disease (GLILD)	18 (12.7)
Autoimmune diseases	16 (11.3)
● Immune thrombocytopenia (ITP)	9 (6.3)
● Hashimoto’s disease	2 (1.4)
● Psoriasis	1 (0.7)
● Autoimmune hepatitis	1 (0.7)
● Atopic dermatitis	1 (0.7)
Chronic spontaneous urticaria (CSU)	2 (1.4)
Behcet’s syndrome	1 (0.7)

**Table 3:** Lymphocyte count and immunoglobulin levels at diagnosis.

Analysed Variables	IEI Patients (*n* = 141)
*Leukocyte count*	
WBC (cells/mm^3^)	5853.70 ± 2226.51
Total lymphocytes (cells/mm^3^)	1893.61 ± 1026.82
T cells (cells/mm^3^)	1536.51 ± 939.48
CD4+ T cells (cells/mm^3^)	893.28 ± 904.98
CD19+ B cells (cells/mm^3^)	160.46 ± 134.87
SM B cells (%)	8.19 ± 9.74
*CD21^low^* B cells (%)	13.53 ± 39.47
*Immunoglobulin levels*	
IgG (mg/dl)	400.60 ± 189.30
IgA (mg/dl)	46.33 ± 49.07
IgM (mg/dl)	50.76 ± 57.78

Table 3—IEI = inborn errors of immunity, WBC = white blood cells; SM B cells = switched memory B cells; IgG = immunoglobulin G; IgA= immunoglobulin A; IgM = immunoglobulin M.

**Table 4:** Macroscopic and microscopic involvement of the colon in IEI patients.

Analysed Variables	IEI Patients (*n* = 141)
*No abnormalities*	*n*= 49 (34.7%)
*Macroscopic findings*	*n*= 79 (56%)
Haemorrhoids	13 (16.5%)
Polyps	42 (53.2%)
Diverticula	5 (6.3%)
Nodularities	4 (5%)
Mucosal oedema	9 (11.4%)
Erosions	6 (7.6%)
*Microscopic*	*n* = 60 (42.5%)
NLH	24 (40%)
Cryptitis	3 (5%)
Crypt abscesses	5 (8.3%)
Lamina propria infiltrate	4 (6.6%)
Collagenous colitis	0 (0%)
Lymphocytic colitis	2 (3.3%)
Acute colitis	3 (5%)
Tubular adenoma	19 (31.7%)
*Leukocyte infiltrate*	*n*= 67 (47.5%)
Neutrophils	9 (13.4%)
Lymphocytes	33 (49%)
Eosinophils	10 (14.9%)
Plasma cells	15 (22.4%)

IEI: inborn errors of immunity; NLH: nodular lymphoid hyperplasia.

**Table 5:** Correlation between GI symptoms and microscopic alterations in the colon.

	GI Symptoms			
Microscopic Alterations in the Colon	*No Symptoms (n = 69; 49.6%)*	*Abdominal Cramps and Diarrhoea (n = 51; 36.7%)*	*Haematochezia (n = 2; 1.4%)*	*Dyspepsia (n = 17; 12.2%)*
*No alterations (n = 80; 57.6%)*	50	22	2	6
*Cryptitis (n = 3; 2.1%)*	0	3	0	0
*Cryptic abscesses (n = 5; 3.6%)*	1	4	0	0
*Lamina propria infiltrate (n = 4; 2.9%)*	1	1	0	2
*Collagenous colitis (n = 0; 0%)*	0	0	0	0
*Lymphocytic colitis (n = 1; 0.7%)*	0	0	0	1
*Acute colitis (n = 3; 2.1%)*	0	0	0	3
*NLH (n = 24; 17.3%)*	8	13	0	3
*Tubular adenomas (n = 19; 13.7%)*	9	8	0	2

Table 5—GI = gastrointestinal; NLH= nodular lymphoid hyperplasia.

**Table 6:** Haematological differences between NLH and non-NLH patients.

		All IEI			CVID	
	NLH (*n* = 24)	No NLH (*n* = 117)	*p*-Value	NLH (*n* = 19)	Non-NLH (*n* = 102)	*p*-Value
***WBC* (*cells*/mm^3^)**	5061.43 ± 1541.58	6023.47 ± 2318.54	0.024	5278.75 ± 1556.29	5997.59 ±2335.05	n.s.
***Lymphocytes* (*cells*/mm^3^)**	1486.93 ± 579.55	1981.65 ± 1082.14	0.005	1561.97 ± 644.35	2024.23 ± 1150.41	n.s.
***CD3+ T cells* (*cells*/mm^3^)**	1234.38 ± 448.85	1618.52 ± 1020.37	0.019	1329.09 ± 489.95	1641.60 ± 1055.68	n.s.
***CD4+ T cells* (*cells*/mm^3^)**	702.30 ± 289.87	944.39 ± 1003.56	n.s.	711.28 ± 330.15	955.59 ± 1053.02	n.s.
***CD19+ B cells* (*cells*/mm^3^)**	180.06 ± 94.16	155.68 ± 143.14	n.s.	200.74 ± 98.90	155.88 ± 144.01	n.s.
***SM B cells (%)***	10.70 ± 8.81	7.39 ± 9.97	n.s.	8.47 ± 7.28	7.47 ± 10.15	n.s.
***CD21^low^ B cells* (*cells*/mm^3^)**	7.18 ± 7.78	15.28 ± 44.30	n.s.	7.73 ± 7.81	15.83 ± 45.13	n.s.
***IgA* (mg/dL)**	42.78 ± 37.91	47.11 ± 51.32	n.s.	28.11 ± 25.40	36.73 ± 33.89	n.s.
***IgM* (mg/dL)**	49.22 ± 60.43	51.11 ± 57.48	n.s.	28.11 ± 23.37	45.78 ± 54.65	n.s.
***IgG* (mg/dL)**	362.04 ± 189.94	409.40 ± 188.98	n.s.	295.22 ± 136.25	389.69 ± 186.25	n.s.
***IgG Trough Level* (mg/dL)**	814.26 ± 143.29	811.24 ± 185.32	n.s.	777.81 ± 101.55	811.39 ± 172.90	n.s.

Table 6—IEI= inborn errors of immunity; CVID = common variable immunodeficiency; NLH = nodular lymphoid hyperplasia; WBC = white blood cells; SM B cells = switched memory B cells; IgG = immunoglobulin G; IgA = immunoglobulin A; IgM = immunoglobulin M.

## Data Availability

Data will be available if requested.
